# Comparative insights into genome signatures of ferric iron oxide- and anode-stimulated *Desulfuromonas* spp. strains

**DOI:** 10.1186/s12864-021-07809-6

**Published:** 2021-06-25

**Authors:** Yong Guo, Tomo Aoyagi, Tomoyuki Hori

**Affiliations:** Environmental Management Research Institute, National Institute of Advanced Industrial Science and Technology (AIST), 16-1 Onogawa, Tsukuba, Ibaraki, 305-8569 Japan

**Keywords:** Comparative genomics, Chemotaxis, Extracellular electron transport, *c*-type cytochrome, Flagellum, Cytochrome *c* oxidase, Transposable element, *Desulfuromonas*

## Abstract

**Background:**

Halotolerant Fe (III) oxide reducers affiliated in the family *Desulfuromonadaceae* are ubiquitous and drive the carbon, nitrogen, sulfur and metal cycles in marine subsurface sediment. Due to their possible application in bioremediation and bioelectrochemical engineering, some of phylogenetically close *Desulfuromonas* spp. strains have been isolated through enrichment with crystalline Fe (III) oxide and anode. The strains isolated using electron acceptors with distinct redox potentials may have different abilities, for instance, of extracellular electron transport, surface recognition and colonization. The objective of this study was to identify the different genomic signatures between the crystalline Fe (III) oxide-stimulated strain AOP6 and the anode-stimulated strains WTL and DDH964 by comparative genome analysis.

**Results:**

The AOP6 genome possessed the flagellar biosynthesis gene cluster, as well as diverse and abundant genes involved in chemotaxis sensory systems and *c*-type cytochromes capable of reduction of electron acceptors with low redox potentials. The WTL and DDH964 genomes lacked the flagellar biosynthesis cluster and exhibited a massive expansion of transposable gene elements that might mediate genome rearrangement, while they were deficient in some of the chemotaxis and cytochrome genes and included the genes for oxygen resistance.

**Conclusions:**

Our results revealed the genomic signatures distinctive for the ferric iron oxide- and anode-stimulated *Desulfuromonas* spp. strains. These findings highlighted the different metabolic abilities, such as extracellular electron transfer and environmental stress resistance, of these phylogenetically close bacterial strains, casting light on genome evolution of the subsurface Fe (III) oxide reducers.

**Supplementary Information:**

The online version contains supplementary material available at 10.1186/s12864-021-07809-6.

## Background

Microorganisms in earth’s crust can change the redox state of extracellular metal oxides by transporting electrons from organic matter degradation [1–4]. These processes not only control the bioavailability of metal ions [5, 6] but also drive the global biogeochemical cycles [7, 8]. Due to the applicability in bioremediation and bioelectrochemical engineering, there have been a lot of efforts to obtain the culture collections of metal-reducing bacteria from various natural environments, such as sediments and soils [9].

The order *Desulfuromonadales* contains abundant resources of metal oxide reducers, in which *Geobacter sulfurreducens* and its relatives affiliated with the family *Geobacteriaceae* have been well studied to reveal the molecular mechanisms underlying the direct electron transfer from microorganisms to crystalline metal oxides [10–16]. To date, it has been proved that some of the specific *c*-type cytochromes can establish a conductive pathway capable of crossing the inner membrane, periplasm and outer membrane that allows the *Geobacter* species to directly transport electrons from the quinone/quinol pool to metal oxides [12, 17]. Additionally, these bacteria produce the electrically conductive protein nanowires, i.e., the e-pili assembled from the PilA pilin monomer, to achieve long-range extracellular electron transport to metal oxide particles [16]. Compared to metal oxides serving as electron acceptor, constant voltage electrodes represent the unlimited electron-accepting potentials allowing microorganisms in contact with the inorganic surface to support the growth of the other distant ones, if these microorganisms can create a conductive network to relay electrons to the electrode [18]. A number of studies showed that some of metal-reducing bacteria utilized the distinctive electron transfer proteins to access the electrodes. Chan and the colleagues constructed the genome-wise transposon-insertion sequencing libraries of *G. sulfurreducens* and found a new putative porin-cytochrome conduit complex (i.e., *extABCD*) crucial for growth with electrodes but not for Fe (III) oxide reduction [19]. Zacharoff et al. reported that deletion of the *pgcA* gene, encoding an extracellular triheme *c*-type cytochrome, in the *G. sulfurreducens* genome generated the mutants unable to transfer electrons to the Fe (III) and Mn (IV) oxides but able to maintain the ability of electrode reduction [20]. These studies suggest that functional diversification of *c*-type cytochromes from *G. sulfurreducens* was responsible for electron transfer to a variety of extracellular electron acceptors, particularly the metal oxides and electrodes.

Cell mobility and extracellular structures (e.g., type-IV pili) are critical for the *Geobacter* physiology, when *Geobacter* spp. are grown with insoluble Fe (III) oxides [21]. The related metabolic processes could be potentially regulated by chemotaxis-like pathways. Various chemotaxis systems and chemo-receptors (e.g., methyl-accepting chemotaxis proteins [MCPs]) have been well characterized in the *Geobacter* genomes and probably play distinct roles in different cellular processes [22]. For example, it is suggested that the *E. coli*-like and *Frz*-like clusters participate in the flagellum-based and social motilities, respectively, while the *Dif*-like and *Deltaproteobacteria*-specific clusters regulate the synthesis of extracellular matrix materials, such as exopolysaccharides [22]. Moreover, the *G. sulfurreducens* mutants lacking components of a methyl-accepting chemotaxis sensing network (i.e., e*snABCD*) were defective in colonization on electrodes but grew normally with Fe (III) oxides, suggesting the distinct recognition and colonization mechanisms between the electrode and Fe (III) oxide reductions [19]. However, such detailed information on the other dissimilatory metal-reducing bacteria is still limited.

Apart from the *Geobacteriaceae* bacteria inhabiting in soils and freshwater ecosystems, the *Desulfuromonadaceae* members, including the genera *Desulfuromonas*, *Pelobacter*, *Desulfuromusa*, *Malomonas* and *Geoalkalibacter*, are mainly isolated from marine sediments [23–26], tidal flat [27], petroleum reservoir [28], other subsurface and/or saline ecosystems [29–31]. *Desulfuromonas* species can catalyze oxidation of a wide range of organic compounds coupled with dissimilatory reduction of metal oxides, elemental sulfur and polysulfide [2, 30, 32]. Due to their high tolerance to saline stress, *Desulfuromonas* spp. have attracted attention for applicability in the saline wastewater treatment [33].

Until recent years, a few of the pure and enrichment cultures of the genus *Desulfuromonas* have been established with different stimulation approaches. ‘*Desulfuromonas soudanensis*’ WTL and *Desulfuromonas* sp. DDH964 were cultured from the underground Soudan iron mine through in situ enrichment with the graphite anode at + 0.24 V (vs the standard hydrogen electrode [SHE]) as electron acceptor [29]. The two strains formed a separate clade together with *Desulfuromonas* sp. TF that was isolated with an electrode as the acceptor [29]. The anode-stimulated strain WTL released electrons at approximately 0.1 V more positive redox potentials compared to *G. sulfurreducens*, while it showed slower Fe (II) generation rates when incubated with a poorly crystalline Fe (III) oxide*,* revealing that its extracellular respiration is tuned for high-potential electron acceptors [29]. The strain WTL survived under the unplanned oxygen exposures, due to power outage in laboratory [29], suggesting the oxygen tolerance ability of this strain. On the other hand, *Desulfuromonas* sp. AOP6 was anaerobically isolated from the subseafloor sediments through enrichment with the crystalline Fe (III) oxide goethite (α-FeOOH) as electron acceptor in our previous studies [34, 35]. The redox potentials of the crystalline Fe (III) oxides (less than − 0.1 V vs SHE) [36] were lower than that of the constant electrode used to isolate the strain WTL (+ 0.24 V vs SHE) [29], suggesting that the crystalline Fe (III) oxide-stimulated strain could use low-potential electron acceptors. In addition, another strict anaerobic strain, *Desulfuromonas acetexigens* 2873^T^, was originally isolated from the digester sludge using acetate and elemental sulfur as the electron donor and acceptor, respectively, corresponding to the redox potential of − 0.24 V vs SHE [32, 37]. This sulfur-stimulated strain possesses a single flagellum in polar and can respire the elemental sulfur [32], as well as transport electron to the anode [38], while its ability of reducing the Fe (III) oxides has not been examined. These *Desulfuromonas* spp. strains obtained using electron acceptors with distinct redox potentials show different physiological properties and thus they may have distinct gene features, for instance, on extracellular electron transport, chemotaxis systems and oxygen tolerance. However, the functional traits and relationships of these phylogenetically close bacterial strains have not been studied well.

The objectives of this study were (i) to determine the genome-wise phylogenetic relationship of all the available genomes from the representative isolates in the order *Desulfuromonadales* (i.e., the higher taxon of the family *Desulfuromonadaceae*) and (ii) to perform comparative genome analysis to identify the different genomic signatures of the phylogenetically close *Desulfuromonas* spp. strains AOP6, WTL and DDH964 that were isolated using the different electron accepters, i.e., crystalline ferric iron oxide and anode.

## Results & discussion

### Phylogenomic position of halotolerant Fe (III) oxide-reducing bacteria in the family *Desulfuromonadaceae*

**Fig. 1:**
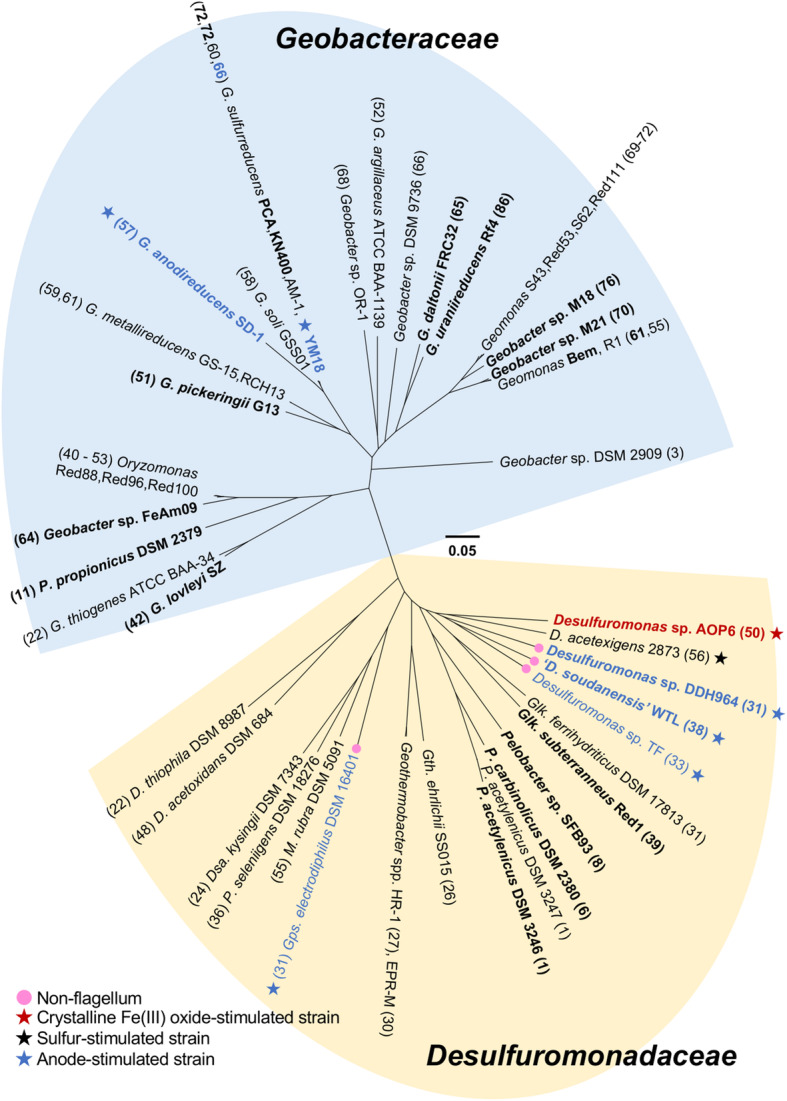
Phylogenomic position of the 5 *Desulfuromonas* spp. strains in the reconstructed maximum likelihood (ML) tree based on the concatenated amino acid sequences deduced from 371 single-copy housekeeping genes. The branch length shows genetic distance with 1000 bootstrap replicates. Blue color font shows the anode-stimulated strains in the order *Desulfuromonadales*. Bold and normal fonts show the complete and incomplete genome sequences, respectively, of the strains. Number in brackets shows the count of deduced multiheme *c*-type cytochrome in the genomes. Pink circle shows the strain that had no gene cluster for flagellum biosynthesis, and the red, black and blue stars show the strains stimulated by using the crystalline Fe (III) oxide, elemental sulfur and anode as the electron acceptors, respectively

A genome-wide phylogenetic analysis of the order *Desulfuromonadales* was performed using concatenated amino acid sequences of 371 conserved proteins from 50 *Desulfuromonadales* genomes. The maximum-likelihood phylogenetic tree showed that these genomes were clearly grouped into two clades (Fig. 1). One clade consisted of the genera within the family *Desulfuromonadaceae*, i.e., *Desulfulomoas*, *Pelobacter*, *Geoalkalibacter*, *Desulfuromusa* and *Malonomonas,* and the two *Geobacteriaceae*-related genera *Geothermobacter* and *Geopsychrobacter* [39], which is defined as the *Desulfuromonadaceae* clade. The other contained the genera within the family *Geobacteraceae*, i.e., *Geobacter*, two recently-described *Geomonas* [40] and *Oryzomonas* [41], and a single species of *Pelobacter propionicus*, which is defined as the *Geobacteraceae* clade. The recently discovered crystalline Fe (III) oxide reducer *Desulfuromonas* sp. AOP6 [34, 35] was placed on the deepest branch of the *Desulfuromonadaceae* clade and formed a cluster together with the sulfur-reducing *Desulfuromonas acetexigens* 2873^T^ [32, 38]*.* The electrode-respiring bacteria ‘*Desulfuromonas soudanensis* WTL’ [29], *Desulfuromonas* sp. DDH964 (or named as ‘*Ca*. Desulfuromonas biiwaabikowi DDH964’ [29]) and *Desulfuromonas* sp. TF [27] were phylogenetically close to each other and formed a cluster neighbor to the AOP6 cluster. These two clusters formed a subclade neighbor to the genus *Geoalkalibacter*.

Despite the phylogenetically close relationship of these Fe (III) oxide-, sulfur- and anode-stimulated bacterial strains AOP6, 2873^T^, WTL, DDH964 and TF, their genome sizes (3.26 to 4.40 Mb) and G + C contents (56.4 to 62.2%) were different to some extent (Table 1). Such differences appeared to be associated with the transposon expansion but not plasmid expansion in the genome. Neither plasmid-like sequence nor plasmid replication gene was found in the genomes of the 5 strains. In addition, the pairwise average nucleotide identities (ANI) among the 5 genomes were in a range from 69.9 to 72.5% (Table 2), being lower than the reported ANI cutoff values of the same species (94 ~ 95%) [42]. Meanwhile, the pairwise average amino acid identities (AAI) of them ranging from 62.7 to 66.5% (Table 2) were lower than the threshold value (i.e., 70%) to define the recently described genera *Geomonas* and *Oryzomonas* in the family *Geobacteraceae* [40, 41]. These results suggested that some of these *Desulfuromonas* spp. strains would represent the new species in the family *Desulfuromonadaceae*.

**Table 1:** *Desulfuromonas* genomes used for comparative genomics

Name	Isolation approach	Sample type	Assembly level	GC%	Size [bp]	CDS	Transposase	Reference	Accession
*Desulfuromonas* sp. AOP6	Geothite stimulated	Cell culture	Complete	56.4	3,269,909	3000	10	Guo et al. 2020	AP022810
*D. acetexigens* 2873^T^	Sulfur stimulated	Cell culture	Contig	60.3	3,683,125	3388	31	Katuri et al. 2017	GCF_900111775
‘*D. soudanensis* WTL’	Anode stimulated	Cell culture	Complete	61.2	3,958,620	3504	78	Badalamenti et al. 2016	CP010802
*Desulfuromonas* sp. DDH964	Anode stimulated	Enrichment	Complete	62.2	3,924,652	3574	111	Badalamenti, JP (unpublished)	CP015080
*Desulfuromonas* sp. TF	Electrode stimulated	Enrichment	Scaffold	58.6	4,402,753	4013	6	Kim et al. 2014	GCF_000472285

**Table 2:** Pairwise similarities (%) of ANI and AAI among the five *Desulfuromonas* genomes

		ANI				
		AOP6	2873^T^	WTL	DDH964	TF
AAI	AOP6	*	70.4	70.6	70.1	69.9
	2873^T^	63.5	*	72.2	71.6	71.0
	WTL	64.7	63.8	*	72.3	72.5
	DDH964	63.3	62.8	65.3	*	71.0
	TF	63.9	62.7	66.5	64.7	*

### General comparison of crystalline Fe (III) oxide- and anode-stimulated *Desulfuromonas* spp. strains

**Fig. 2:**
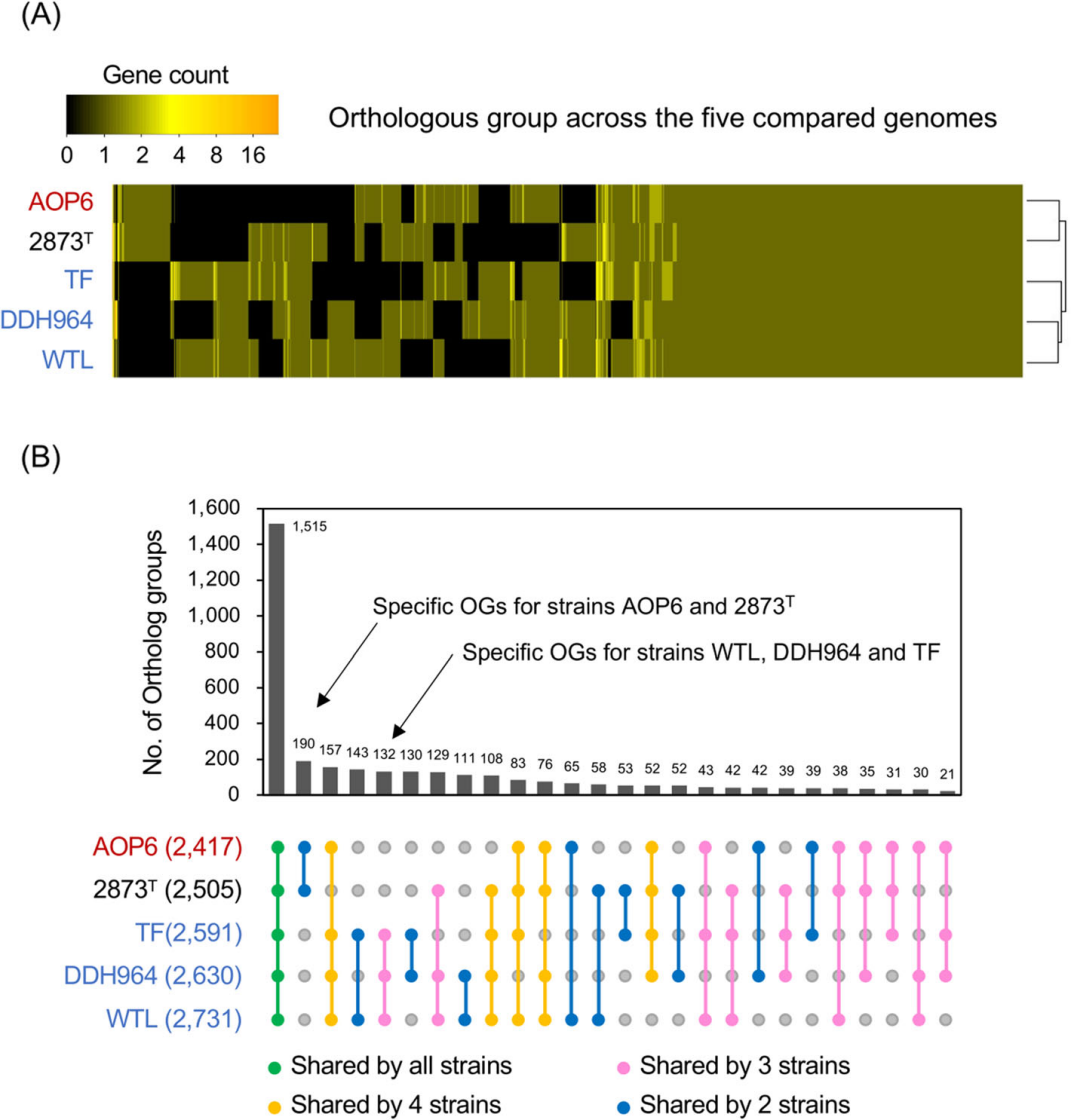
Orthologous groups (OGs) across the genomes of the 5 *Desulfuromonas* spp. strains. A, heatmap with hierarchical clustering showing the OGs patterns of the 5 strains. B, bar plot showing the counts of OGs shared by the different strains. Red-, black- and blue-colored strain names in A and B show those strains stimulated by using the crystalline Fe (III) oxide, elemental sulfur and anode as the electron acceptors, respectively. Number above each bar in B shows the count of OGs shared by the strains. Number in brackets in B shows the count of OGs deduced in the genome of each strain. The colored dots with links in B show the orthologous groups shared by all the 5 strains (green), 4 of the strains (yellow), 3 of the strains (pink), and 2 of the strains (blue)

A phylogenetic orthology analysis using OrthoFinder [43] was performed to compare the genomes of the 5 *Desulfuromonas* spp. strains. Overall, 14,437 of the total 17,479 genes from the compared 5 genomes, accounting for 82.6% of the total genes, were assigned into 3423 orthologous groups (OGs) (Fig. 2). Hierarchical clustering analysis of the OGs revealed the different patterns of gene contents among the Fe (III) oxide-, sulfur-, and anode-stimulated strains (Fig. 2A). Among these OGs, 1515 OGs were identified as the core OGs, ranging from 42.1 to 54.8% of the total genetic elements of each strain (Fig. 2B). In addition, 190 and 132 OGs were shared only by the strains isolated using the inorganic electron acceptors (i.e., ferric iron oxide or sulfur) and anode, respectively (Fig. 2B). These results underscored a large genetic difference of these phylogenetically close bacterial strains.

**Fig. 3:**
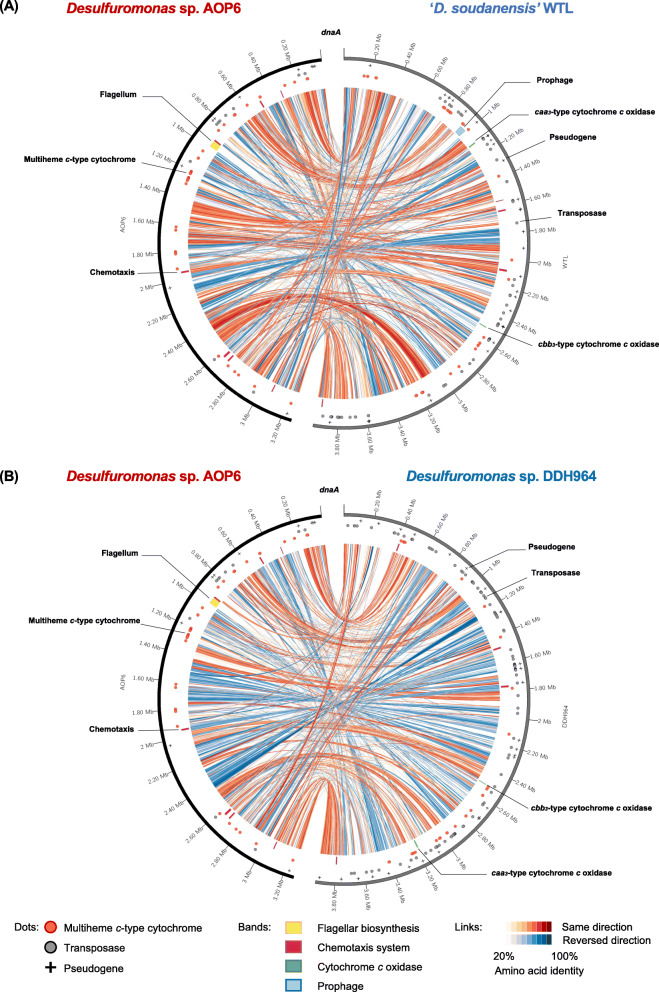
Syntenic comparative genomic analysis data using the complete genome sequences of the strains AOP6, WTL and DDH964. A, the strain AOP6 versus WTL. B, the strain AOP6 versus DDH964. Red- and blue-colored strain names in A and B show those strains stimulated by using the crystalline Fe (III) oxide and anode as the electron acceptors, respectively. Symbols next to the scale show the positions of pseudogenes (black crosses), as well as those of the genes responsible for the biosyntheses of the multiheme *c*-type cytochromes (red dots) and transponsases (grey dots), while bands next to the links show the positions of gene clusters responsible for the biosyntheses of the flagellum (yellow), chemotaxis system (magenta) and cytochrome *c* oxidase (green), as well as that of the prophage (sky blue). Links associated with the two genomes show homologous genes in same (red) and reverse (blue) directions. The gradient color of the link shows the identity between the amino acid sequences deduced for the homologous genes

Syntenic comparisons of the complete genome sequence of the Fe (III) oxide-stimulated strain AOP6 with those of the anode-stimulated strains WTL (Fig. 3A) and DDH964 (Fig. 3B) were performed to determine the genomic difference and rearrangement between the *Desulfuromonas* spp. strains stimulated with different electron acceptors. From the comparisons, the Fe (III) oxide-stimulated strain AOP6 was characterized by the presence of a complete gene cluster for flagellar biosynthesis in the genome (Fig. 3). Interestingly, this gene cluster was highly conserved in the genomes of *Desulfuromonadales* bacteria except for 4 strains of the anode-stimulated *Desulfuromonadaceae* bacteria (i.e., ‘*D. soudanensis*’ WTL, *Desulfuromonas* sp. DDH964, *Desulfuromonas* sp. TF, and *Geopsychrobacter electrodiphilus* DSM 16401) (Fig. 1). Previous studies revealed that *Geobacter* species expressed flagella for chemotaxis toward Fe (III) oxides in subsurface environments to achieve the efficient respiration [21, 44]. The conservation of the flagellar biosynthetic gene cluster by the *Desulfuromonadales* members may have reflected their long-term association with the Fe (III)-oxide respiration, because Fe (III) has been thought to be abundant on the primitive Earth, potentially serving as the globally significant extracellular electron acceptor, even prior to the availability of nitrate, sulfate, and oxygen [45]. However, in the possible evolutionary history of *G. sulfurreducens*, a transposase gene was inserted into the master transcriptional regulator for the flagellar gene expression, which disrupted the flagella expression and further impaired the Fe (III) oxide reduction [44]. Therefore, we hypothesized that the lack of the flagellar biosynthetic gene cluster would trigger or enhance the Fe (III) oxide-reducing bacteria to alter the way to transfer electrons, e.g., to use alternative electron acceptors. Our comparative analysis showed that the AOP6 genome contained more chemotaxis gene clusters and specific multiheme *c*-type cytochrome encoding genes, whereas the WTL and DDH964 genomes possessed the operons for the *caa*_*3*_- and *cbb*_*3*_-type cytochrome *c* oxidases, more abundant transposable elements (TEs), and more abundant pseudogenes. Additionally, a number of genomic rearrangements identified from the comparisons (the strain AOP6 vs WTL or DDH964) were widely distributed across the whole genomes (Fig. 3). The genomic rearrangement likely affected the gene expression and induced the loss of gene function when a breakpoint occurred inside a reading frame [46, 47]. Actually, 34 and 27 pseudogenes predicted in the genomes of the anode-stimulated strains WTL and DDH964 were identified by frameshifting and missing the N- and/or C-terminus (data not shown). Taken together, these differences highlighted the functional diversification of the dissimilatory reduction processes catalyzed by the ferric iron oxide- and anode-stimulated *Desulfuromonas* spp. strains.

### Chemotaxis system

**Fig. 4:**
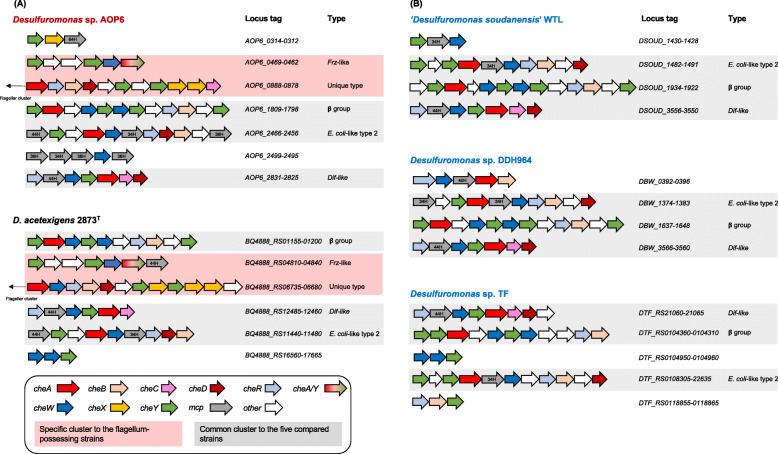
Physical arrangements of the chemotaxis gene clusters in the complete genomes of the 5 strains. A, the gene clusters possessed by the crystalline Fe (III) oxide- and sulfur-stimulated strains; B, the gene clusters possessed by the anode-stimulated strains. Red- and black-colored strain names in A as well as the blue-colored strain names in B show those strains stimulated by using the crystalline Fe (III) oxide, sulfur and anode as the electron acceptors, respectively. Different colors show the chemotaxis genes of *cheA* (red) coding for an autophosphorylating histidine kinase, *cheB* (champagne) for a methylesterase, *cheC* (pink) for a protein phosphatase, *cheD* (maroon) for a chemoreceptor glutamine deamidase, *cheR* (baby blue) for a methyltransferase, *cheW* (blue) for a scaffold protein, *cheX* (yellow) for a dimer-form protein phosphatase, *cheY* (green) for a response regulator, and *cheA/Y* (a gradient from red to green) for a CheA-CheY fusion protein, as well as *mcp* (grey) coding for a methyl-accepting chemotaxis protein and other genes (white). Locus tags and types of chemotaxis clusters are listed next to the diagrams. The types of chemotaxis clusters are identified by comparing with the well-known 6 types of chemotaxis clusters conserved in the genomes of *Geobacter sulfurreducens*, *Geobacter metallireducens* and *Geobacter uraniireducens*. *E. coli*-like type 2 contains the non-chemotaxis genes and multiple *mcp* genes in the cluster. *Frz*-like type contains the *cheA/Y* fusion genes in the cluster. *Dif*-like type contains the genes of 44H-*mcp, cheA*, *cheC*, *cheD*, *cheW* and *cheY* in the cluster. *Deltaproteobacteria*-specific β group clusters contain the genes of *cheA*, *cheB*, *cheR* and *cheW*, but no *mcp* genes. Red-colored background of the gene clusters indicates the specific clusters to the flagellum-possessing strains AOP6 and 2873^T^, while grey-colored background indicates the common clusters to the 5 strains

Multiple copies of the chemotaxis genes were identified in the 5 genomes by using Microbial Signal Transduction Database (MiST) 3.0 [48]. Despite relatively small sizes of these genomes, 61 and 64 homologs to the *che* and *mcp* genes were found in the strains AOP6 and 2873^T^ genomes, respectively, whereas the strains WTL, DDH964 and TF possessed 48 to 56 of these genes (Table 3). Most of the *che* genes were clustered in the genomes of the 5 strains (Fig. 4), except for the genes for chemoreceptors of the MCPs that were dispersed throughout the genomes (data not shown). The organization pattern of chemotaxis genes was consistent with those found in the genomes of *Geobacter* species [22]. There were 7, 6, 4, 4 and 5 major chemotaxis gene clusters in the AOP6, 2873^T^, WTL, DDH964 and TF genome, respectively. Their physical arrangements are showed in Fig. 4. In the case of the strains AOP6 and 2873^T^, the chemotaxis gene clusters (locus tags: AOP6_0888–0878 and BQ4888_RS06735–06680) were located on the upstream of the flagellar biosynthesis gene clusters (AOP6_0942–0890 and BQ4888_RS06745–06865).

**Table 3:** Gene counts of chemotaxis homologs in the five *Desulfuromonas* genomes

		*Desulfuromonas*				
		AOP6	2873^T^	WTL	DDH964	TF
*cheA*		4	5	3	4	3
*cheAY* ^*a*^		1	1	0	0	0
*cheB*		3	3	4	4	6
*cheR*		3	5	3	4	6
*cheW*		6	8	9	6	8
*cheX* ^*a*^		3	3	0	0	0
*cheY* ^*b*^		15 (11)	16 (10)	17 (8)	10 (6)	21 (8)
*cheC*		2	2	1	1	2
*cheD*		3	2	2	2	2
*cheV*		0	1	0	0	1
*mcp*	Total	14	18	15	10	7
	34H	2	3	8	2	1
	36H	5	7	0	0	0
	40H	2	2	1	3	1
	44H	4	5	4	2	3
	64H	1	1	2	3	2
Total ^*c*^		61 (46)	64 (40)	54 (27)	48 (29)	56 (29)
*che* cluster		7	6	4	4	5

^*a*^The specific *che* genes possessed by the flagellum-harboring strains AOP6 and 2873^T^

^*b*^The numbers in brackets indicate the counts of *cheY* genes in the major clusters shown in Fig. 4

^*c*^The numbers in brackets indicate the counts of chemotaxis genes in the major clusters shown in Fig. 4

Three to 6 of the *cheA* and *cheAY* genes, encoding the autophosphorylating histidine kinase (CheA), were predicted to be on the 5 genomes (Table 3), while these were homologous to the known *cheA* genes in the *Geobacter* species. Previously, Tran and the colleagues reported that the multiple *cheA* genes in the *Geobacter* species were the paralogous genes that evolved separately, whereas each of *cheA* gene together with other *che* genes likely regulated a separate chemotaxis pathway [22]. Moreover, 10 to 21 of the *cheY* genes, encoding response regulators as the substrates of CheA-mediated phosphorylation in chemotaxis pathways, were predicted to be on the 5 genomes. But only 6 to 11 of these genes were arranged in the chemotaxis gene clusters, being probably involved in chemotaxis signaling [49]. Meanwhile, the remainder *cheY* genes were located on the chromosome, thus some of them seemed to function in the two-component pathways unrelated to chemotaxis [22]. These two-component pathway-related *cheY* genes were detected only in the genomes of the strains AOP6 and 2873^T^.

A number of genes for MCPs were found in the 5 genomes; 14 in the strain AOP6, 18 in 2873^T^, 15 in WTL, 10 in DDH964, and 7 in TF (Table 3). MCPs have been assigned to different classes that designated 24H, 28H, 34H, 36H, 38H, 40H, 44H and 64H according to the number of 7-aa heptad repeats (H) in the cytoplasmic domain [50]. Among them, the MCPs of the classes 36H and 44H were well characterized in *Escherichia coli* and *Bacillus subtilis*, respectively. In the class of 36H receptors of *E. coli*, the positive stimuli increased methylation at all the responsible sites and further decreased kinase activity, whereas the class 44H receptor of *B. subtilis* had a different methylation mechanism, in which only one site was methylated and the other sites were demethylated in response to the positive stimuli, resulting in the increase in kinase activity [51]. Multiple alignments of the predicted MCPs showed that the strains AOP6 and 2873^T^ had MCPs of the classes 34H, 36H, 40H, 44H and 64H, and the strains WTL, DDH964 and TF had those of the classes 34H, 40H, 44H and 64H. Although the anode-stimulated strains lacked the MCPs of the class 36H that were previously detected in the genomes of *Geobacter metallireducens* and *G. uraniireducens* [22], all of these 5 strains possessed the homologs of the 40H-MCP EsnA that was probably associated with the colonization on electrodes [19].

Six types of the chemotaxis clusters in the *G. sulfurreducens*, *G. metallireducens* and *G. uraniireducens* genomes were well characterized [22]. Here, the types of chemotaxis clusters found in the 5 genomes were predicted by comparison with the above-mentioned 6 types. The *E. coli*-like cluster type 2, *Dif*-like and *Deltaproteobacteria*-specific β group clusters were found in all the genomes (Fig. 4); whereas the *Frz*-like clusters were detected in the AOP6 and 2873^T^ genomes (Fig. 4). Though the chemotaxis clusters AOP6_0888–0878 and BQ4888_RS06735–06680 in the upstream of the flagellar clusters were similar to the β group clusters containing the chemotaxis genes (i.e., *cheA*, *cheW*, *cheB* and *cheR*) but no *mcp* genes, this cluster also included other chemotaxis genes (i.e., *cheC*, *cheD* and *cheX*), indicating a unique type of chemotaxis cluster for the strains AOP6 and 2873^T^ (Fig. 4A). The *cheC* and *cheD* genes, encoding the phosphatases of CheY protein (CheY-P) and the chemoreceptor glutamine deamidases, respectively, were translationally coupled and interacted to each other to regulate the chemotaxis sensing, whereas the *cheX* gene encoded another type of CheY-P phosphatase, i.e., CheX, that was different from the CheC [52]. Two monomers of CheX formed a homodimer as a functional unit that represented the most powerfulness CheC-type phosphatase [52, 53]. Due to the physically position neighbor to the flagellar clusters, it was tempting to speculate that these clusters (AOP6_0888–0878 and BQ4888_RS06735–06680) regulated the flagellar-based motility of the strains AOP6 and 2873^T^. However, as the complexity of gene organization, the functional details of this cluster should be further examined by molecular approaches. Overall, compared with the anode-stimulated *Desulfuromonas* spp. strains, the Fe (III) oxide- and sulfur-stimulated strains AOP6 and 2873^T^ possessed more abundant and diverse chemotaxis genes, in addition to the complete flagellar biosynthesis gene cluster, thereby enabling their possibly versatile cellular behaviors in response to environmental stimuli.

### Multiheme *c*-type cytochrome

**Fig. 5:**
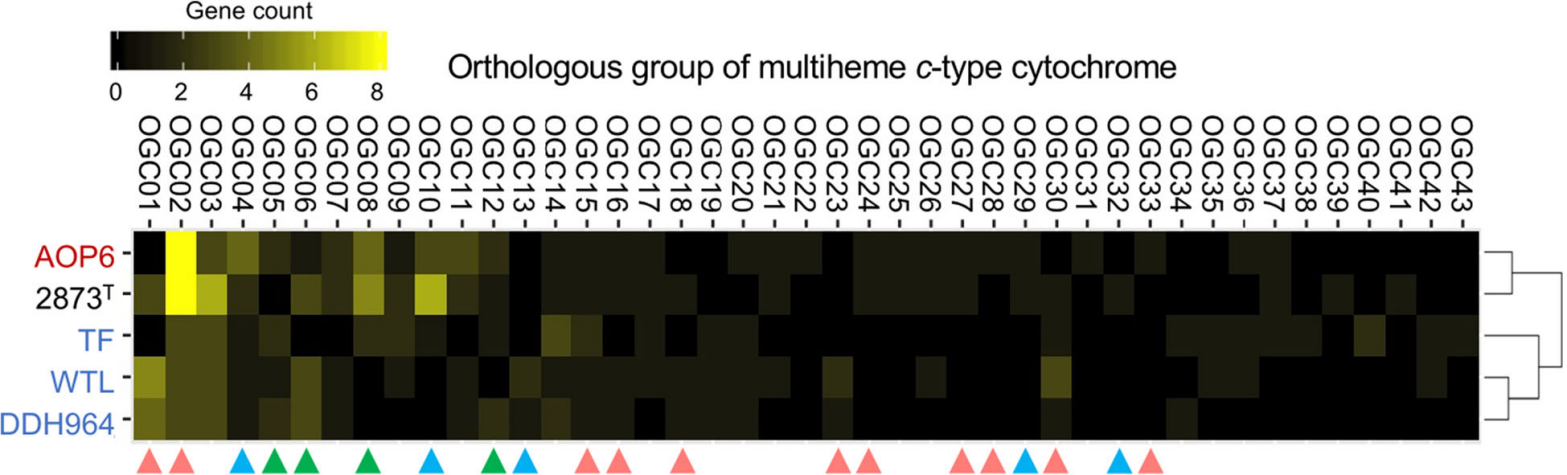
Heatmap with hierarchical clustering showing the orthologous groups of multiheme *c*-type cytochromes (OGC) of the 5 *Desulfuromonas* spp. strains. Red-, black-, and blue-colored strain names show those strains stimulated by using the crystalline Fe (III) oxide, sulfur and anode as the electron acceptors, respectively. Triangles point out the multiheme *c*-type cytochromes reported to be relevant to the electron transport to Fe (III) oxide (red), anode (blue) and both the Fe (III) oxide and anode (green)

Multiheme *c*-type cytochromes are key for electron transport during the metal oxide and electrode respirations. To assess the diversity of the cytochromes, 28, 27, 24, 19 and 22 orthologous groups of multiheme *c*-type cytochromes (OGCs) were picked up from the genomes of the strains AOP6, 2873^T^, WTL, DDH964 and TF, respectively (43 OGCs in total). A heatmap with hierarchical clustering of these OGCs showed that the profiles of multiheme *c*-type cytochrome of the 5 strains were clearly grouped into two clusters; one consisted of the strains AOP6 and 2873^T^, while the other was comprised of the strains WTL, DDH964 and TF (Fig. 5). There were only 5 core OGCs possessed by all the five strains, i.e., the homologous genes of *omcI* (OGC02), *ppcA* (OGC03), *omcQ* (OGC04), *imcH* (OGC14) and *cbcL* (OGC15) characterized in the *Geobacter* species [10, 54]. ImcH and CbcL were identified as the inner membrane *c*-type cytochromes involved in the early steps of electron transfer to extracellular substrates in *G. sulfurreducens*, and further the two cytochromes would be required for reduction of electron acceptors with different redox potentials [55]. ImcH was for the acceptors with redox potentials higher than 0.1 V vs SHE, such as Fe (III) citrate and insoluble Mn (IV) oxides [56], while CbcL was for the low redox potential acceptors, such as the crystalline Fe (III) oxide goethite [57]. PpcA, a periplasmic *c*-type cytochrome, was employed in reduction of Fe (III) citrate [58], but not insoluble Fe (III) oxides and electrodes [59]. OmcI, an outer membrane *c*-type cytochrome, appeared to participate in reduction of Fe (III) oxide [59], while another one, i.e., OmcQ, was likely irrelevant to reduction of Fe (III) oxide [60], despite the fact that the OmcQ expression significantly increased when cultivated under a low electrode potential of − 0.25 V vs SHE [61]. Although the 5 core OGCs of the *c*-type cytochrome genes were included in the 5 *Desulfuromonas* genomes in this study, the AOP6 and 2873^T^ genomes conserved more abundant homologous genes of *omcI* than the other genomes. In contrast, the anode-stimulated strains seemed to possess multiple copies of *imcH* (2, 3 and 4 copies for the strains WTL, DDH964 and TF, respectively). Despite the presence of commonly conserved *c*-type cytochromes, a large number of OGCs specific to these strains may have formed distinct electron transport ways to different electron acceptors (Fig. 5).

Previous studies revealed that the porin cytochrome conduit (Pcc) complexes acted as conduits for transporting electron across the outer membrane in the *Geobacter* species, which consisted of the periplasmic multiheme *c*-type cytochromes, porin-like outer membrane protein and outer membrane *c*-type cytochrome [62, 63]. In the case of *G. sulfurreducens*, the putative Pcc complexs *ombB-omaB-omcB* were needed for electron transport to Fe (III) oxides [59, 64]. OmcB, an outer membrane lipoprotein *c*-type cytochrome of the *ombB-omaB-omcB* complex, was thought to transfer electrons to the terminal reductases, such as extracellular multiheme *c*-type cytochromes [62, 65]. Additionally, the homolog of OmcB in *Desulfuromonas acetoxidans* was able to directly bind to the Ag electrode and promoted electron transport at the biofilm-electrode interface [66]. In this study, the *Desulfuromonas* spp. strains AOP6, DDH964 and TF possessed the complete gene cluster for the *ombB-omaB-omcB* complex (data not shown), while the strains 2873^T^ and WTL lacked the *omcB* (OGC05) and *ombB* (OGC12) genes, respectively (Fig. 5). Another Pcc complex *extABCD* might be required for electrode respiration but not for Fe (III) oxide reduction [19]. The genomes of the strains AOP6 and 2873^T^ possessed the homologous genes for *extA* (OGC10) encoding the periplasmic cytochrome *c*, while the WTL and DDH964 genomes possessed the *extC* homologs (OGC13) encoding the outer membrane cytochrome *c*; however, none of the 5 strains possessed the complete *extABCD* complex gene cluster. Some of singleton genes for other periplasmic cytochrome *c* of certain Pcc complex were also found in the genomes of the 5 strains: *pccF* (OGC07) in the strains AOP6, 2873 T, WTL and DDH943, and *extK* (OGC29) in the strains AOP6 and 2873^T^.

Other genes encoding the outer membrane *c*-type cytochromes, such as *omcO* (OGC01), *omcP* (OGC06), *omcS* (OGC08) and *omcZ* (OGC32), were found in the 5 genomes (Fig. 5). OmcO and OmcP appeared to be irrelevant to Fe (III) oxide reduction by *G. sulfurreducens* [59], but their homologs were relevant to that by *G. metallireducens* [67], indicating that these *c*-type cytochromes may have played different functional roles in these species. In this study, the anode-stimulated strains WTL and DDH964 possessed multiple copies of the *omcO* and *omcP* genes, but the ferric iron oxide-stimulated AOP6 possessed only one copy of *omcP*. Meanwhile, OmcS required for both Fe (III) oxide reduction [65] and electrode respiration [68] bound to e-pili as catalyzing the terminal electron transfer [69]. Recent studies showed that OmcS also formed the cytochrome-based filaments capable of transporting electron for long distances over micrometers [14, 15]. Here, multiple homologs of OmcS were found in the strains AOP6, 2873^T^ and TF, but not WTL and DDH964. Further, OmcZ was reported to participate in electron transport to anode with thick biofilm (> 50 μm) [70], but not to anode with thin biofilm and Fe (III) oxides by *G. sulfurreducens* [59, 68]. Only one gene copy encoding the OmcZ homolog was found in the 2873^T^ genome.

Although the strains WTL and DDH964 did not have the homologs of OmcS and OmcZ, they possessed *pgcA* (OGC23) and *gscA* (OGC30) genes encoding extracellular multiheme *c*-type cytochromes that can serve as terminal reductases [71, 72]. PgcA, a soluble c-type cytochrome, was secreted to environments as electron shuttle [71]. The *G. sulfurreducens* mutants with deletion of *pgcA* lost the ability to transfer electrons to the Fe (III) and Mn (IV) oxides, but the same mutants maintained the electrode respiration capability [20], suggesting the enhancement of the metal reductions rather than electrode respiration by PgcA. However, the homolog of PgcA was not found in the genomes of the Fe (III) oxide-stimulated strain AOP6 and sulfide-stimulated strain 2873^T^, but found in the anode-stimulated strains WTL and DDH964. In this context, this gene was also present in the genomes of *G. anodireducens* SD-1 and *G. sulfureducens* YM18 that were isolated from an anode biofilm and generated high current densities in bioelectrochemical systems [72, 73].

Menaquinol:ferricytochrome *c* oxidoreductase (Cbc) complexes, consisting of periplasmic *c*-type cytochromes, *b*-type cytochromes, membrane proteins and other *c*-type cytochromes, were also associated with reduction of the Fe (III) and Mn (IV) oxides in the *Geobacter* species [59]. In this study, the genes *cbcA* (OGC17), *cbcM* (OGC21), *cbcN* (OGC24), *cbcR* (OGC25), *cbcS* (OGC19), and *cbcX* (OGC20) encoding the *c*-type cytochromes of Cbc complexes were found in the 5 genomes (Fig. 5). Reportedly, the expressions of the *cbcA*, *cbcN* and *cbcX* genes for the periplasmic *c*-type cytochromes were up-regulated in *G. sulfurreducens* with Fe (III) oxide versus Fe (III) citrate [59]. It was noteworthy that only the Fe (III) oxide-stimulated strain AOP6 possessed all of these three genes.

A number of other genes encoding *c*-type cytochromes found in the 5 genomes were reported to be significantly up-regulated when Fe (III) oxide was used as electron acceptor, which includes the *nrfA* (OGC09), *nrfH* (OGC26) genes in *G. sulfurreducens* [59], as well as the unnamed genes Gmet_1867 (OGC16), Gmet_0142 (OGC18) and Gmet_0679 (OGC27) in *G. metallireducens* [67]. However, no further evidence can support an association between the arrangements of these genes and the Fe (III) oxide reduction activity. In addition, the other genes for *c*-type cytochromes (OGC31 and OGC33–43) were also present in some of the 5 genomes (Fig. 5). The homologs of these genes were almost found in the genomes of the *Desulfuromonas* species, but absent in those of the *Geobacter* species (data not shown). Their roles in extracellular electron transport warrant the future study. Consequently, our analysis indicated that the multiheme *c*-type cytochrome profiles were obviously different among the phylogenetically close *Desulfuromonas* spp. strains. The Fe (III)-oxide stimulated strains possessed more abundant and diverse *c*-type cytochromes than the anode-stimulated strains. Particularly, the periplasmic and outer membrane cytochromes represented strain- and/or cluster-specific patterns, which may have linked with electron transports to the iron (III) oxide and anode with different redox potentials.

### Cytochrome oxidase and ROS detoxifying enzyme

Although most of members in the families *Geobaceteraceae* and *Desulfuromonadaceae* are strictly anaerobic [37, 74], some of them show the ability to detoxify reactive oxygen species (ROS) and resist low concentrations (e.g., ~ 10%) of oxygen [27, 29, 75, 76]. *G. sulfurreducens* sigma factor RpoS contributed to survival in stationary phase and upon oxygen exposure [75]. RpoS activated the *caa*_*3*_-type cytochrome *c* oxidase operon, which encodes a heme-copper terminal oxidase for the *G. sulfurreducens* growth using oxygen as an electron acceptor [77]. The *caa3*-type oxidase encoded by *coxABCD* was found in the genomes of *G. sulfurreducens* [10], *G. metallireducens* [54], *Geomonas bemidjiensis* (previously named as *Geobacter bemijiensis*) [40, 78], and *Desulfuromonas* sp. TF [27]. On the other hand, the *cbb*_*3*_-type cytochrome *c* oxidase (CcoNOPQ) with high affinity for oxygen was expressed under low oxygen tension conditions [79], and the genes encoding this oxidase were conserved in the genomes of *Gm. bemidjirensis* and *Desulfuromonas* sp. TF [27, 78].

Intriguingly, in this study, the gene sets for these two types of oxidases were present in the genomes of the strains WTL, DDH964 and TF, but absent in those of the strains AOP6 and 2873^T^ except for the homologous genes of *ccoP* that encodes the diheme cytochrome *c* subunit (subunit III) of *cbb*_*3*_-type oxidase (Table 4). Moreover, the anode-stimulated strains possessed multiple homologs of CcoP: 6 in the strain WTL, 4 in DDH964, and 4 in TF. *Pseudomonas aeruginosa*, a ubiquitous opportunistic human pathogen, expressed multiple *cbb*_*3*_-type cytochrome *c* oxidase isoforms by combinations of multiple isosubunits, and the strains carrying these isoforms can resist to respiratory inhibitors such as nitrite and cyanide at low concentrations of oxygen [80]. Despite only one *ccoN* gene encoding the catalytic subunit possessed by the anode-stimulated strains, there was still possibility that they produced 4 to 6 *cbb*_*3*_ isoforms by combinations with different CcoP isosubunits. Such multiple *ccb*_*3*_ isoforms may have brought a diverse array for oxidative stress responses for the anode-stimulated strains. Additionally, the *cydAB* genes encoding cytochrome *bd* quinol oxidase complex was found in the 5 genomes (Table 4), which is related to the provision of the trace oxygen tolerance [27]. Genes involved in the ROS detoxification, e.g., the genes for rubrerythrin (*rbr*), rubredoxin (*rub*), desulfoferredoxin (*dfx*), and cytochrome *c* peroxidase (*macA*), abundantly existed in the 5 genomes (Table 4). It was reported that these genes were significantly expressed in *G. uraniireducens* exposed to 5% oxygen revealed by microarray analysis [81]. Overall, comparative genome analysis showed that the *caa*_*3*_- and *cbb*_*3*_-type cytochrome *c* oxidases appeared to be specific to the anode-stimulated strains WTL, DDH964 and TF. They also possessed other diverse genes involved in oxygen resistance and ROS detoxification. These genetic features would confer high oxygen resistance in the strictly anaerobic bacteria, contributing to the niche differentiation of the phylogenetically close *Desulfuromonas* spp. strains. Reportedly, the anode-stimulated strain WTL originally inhabited in the anoxic (but not strictly anaerobic) environments at the Soudan Iron Mine borehole and it was able to survive under the unplanned oxygen exposures, due to power outage in laboratory [29], which partially supports the genomic inference.

**Table 4:** Genes encoding cytochrome *c* oxidases, cytochrome *bd* oxidase, and ROS detoxifying enzymes in the five *Desulfuromonas* genomes

		Locus tag in the genomes of *Desulfuromonas spp.* strains				
Gene	Product	AOP6	2873^T^	WTL	DDH964	TF
*sco*	*caa*_*3*_ cytochrome *c* oxidase synthesis factor	–	–	DSOUD_0978	DBW_3007	DTF_RS0106035
*coxA*	*caa*_*3*_ cytochrome *c* oxidase, subunit I	–	–	DSOUD_0979	DBW_3008	DTF_RS0106040
*coxB*	*caa*_*3*_ cytochrome *c* oxidase, subunit II	–	–	DSOUD_0982	DBW_3011	DTF_RS0106055
*coxC*	*caa*_*3*_ cytochrome *c* oxidase, subunit III	–	–	DSOUD_0980	DBW_3009	DTF_RS0106045
*coxD*	*caa*_*3*_ cytochrome *c* oxidase, subunit IV	–	–	DSOUD_0981	DBW_3010	DTF_RS0106050
*ctaB*	Proteheme IX farnesyltransferase	–	–	DSOUD_0983	DBW_3012	DTF_RS25235
–	Rubrerythrin	AOP6_2408	BQ4888_RS16015	DSOUD_0984	DBW_3013	DTF_RS0102535
*dfx*	Desulfoferredoxin (superoxide reductase)	AOP6_2409	BQ4888_RS16020	DSOUD_0985	DBW_3014	–
*ccoN*	*cbb*_*3*_ cytochrome *c* oxidase, subunit I	–	–	DSOUD_2312	DBW_2371	DTF_RS0116565
*ccoO*	*cbb*_*3*_ cytochrome *c* oxidase, subunit II	–	–	DSOUD_2313	DBW_2372	DTF_RS0116570
*ccoP*	*cbb*_*3*_ cytochrome *c* oxidase, subunit III	AOP6_0637	BQ4888_RS14340	DSOUD_2315	DBW_2374	DTF_RS26690
*ccoQ*	*cbb*_*3*_ cytochrome *c* oxidase assembly chaperone	–	–	DSOUD_2314	DBW_2373	DTF_RS26685
*ccoS*	*cbb*_*3*_ cytochrome *c* oxidase assembly protein CcoS	–	–	DSOUD_2319	DBW_2378	DTF_RS0116600
–	*cbb*_*3*_ cytochrome *c* oxidase, subunit III	AOP6_0462, AOP6_1193, AOP6_1768, AOP6_2692	BQ4888_RS04845, BQ4888_RS07140	DSOUD_0601	DBW_0838	DTF_RS0119470
–	*cbb*_*3*_ cytochrome *c* oxidase, subunit III	–	–	DSOUD_0719	–	–
–	*cbb*_*3*_ cytochrome *c* oxidase, subunit III	–	BQ4888_RS10570	DSOUD_0327, DSOUD_1618	DBW_3541	DTF_RS25165
–	*cbb*_*3*_ cytochrome *c* oxidase, subunit III	AOP6_1494	–	–	–	–
–	*cbb*_*3*_ cytochrome *c* oxidase, subunit III	AOP6_2835	–	DSOUD_0081	DBW_3572	DTF_RS0100735
*cydA*	cytochrome *bd* ubiquinol oxidase subunit I	AOP6_2873	BQ4888_RS11725	DSOUD_0336	DBW_0564	DTF_RS22095
*cydB*	cytochrome *bd* ubiquinol oxidase subunit II	AOP6_2874	BQ4888_RS11720	DSOUD_0335	DBW_0565	DTF_RS0105770
*rub*	Rubredoxin	AOP6_0414, AOP6_0415	BQ4888_RS07685	DSOUD_3135	DBW_3237	DTF_RS25905
*–*	Desulfoferredoxin (superoxide reductase)	AOP6_2966	–	DSOUD_0362	DBW_3006	–
*rbr*	Rubrerythrin	AOP6_0339	BQ4888_RS13615	DSOUD_0365	DBW_3641	DTF_RS0115795
*sodA*	Superoxide dismutase	–	BQ4888_RS04500	–	–	–
*macA*	Cytochrome *c* peroxidase	AOP6_0104, AOP6_0558	BQ4888_RS09810, BQ4888_RS15550	DSOUD_0544, DSOUD_1166, DSOUD_2534	DBW_1968	DTF_RS0118780

### Transposable element

Transposable elements (TEs) are usually found in the genomes of bacterial symbionts, which initially undergo massive expansion and then loss accompanied by gene inactivation and decay, genome rearrangement and genome reduction [82–85]. The large genome rearrangement and deletions associated with insertion sequence (IS) expansion enabled the symbiotic bacterium to combat host defenses by changing surface antigens and regulatory circuitry [86, 87]. Similar patterns of this genome evolution were detected in cases of some niche-restricted prokaryotes such as *Sulfolobus solfataricus* and certain *Cyanobacteria* species [88–90].

The anode-stimulated strains WTL and DDH964 possessed large numbers of TEs, particularly transposase-encoding genes that accounted for 2.2 and 3.1% of the total coding sequences in the genomes, respectively, whereas the Fe (III) oxide-stimulated strain AOP6 only contains 10 of these genes in the genome (0.3% of the total) (Fig. 3). In addition, most of the transposase-encoding genes were located nearby the pseudogenes in the WTL and DDH964 genomes (Fig. 3), suggesting that the repeated insertion-deletion of transposons induced the loss of some of functional genes, such as those encoding the transcriptional regulator, RNA methyltransferase, ATPase, polysulfide reductase and methyl-accepting chemotaxis protein (data not shown). Meanwhile, 31 and 6 transposase-encoding genes were also found in the 2873^T^ and TF genomes; however, due to the highly fragmented assemblies of the strains 2873^T^ and TF (consisting of 41 and 167 contigs, respectively), the absolute counts of TEs would be underestimated [84]. Thus, these results only suggest an expansion of TEs in the genomes of the strains WTL and DDH964.

**Fig. 6:**
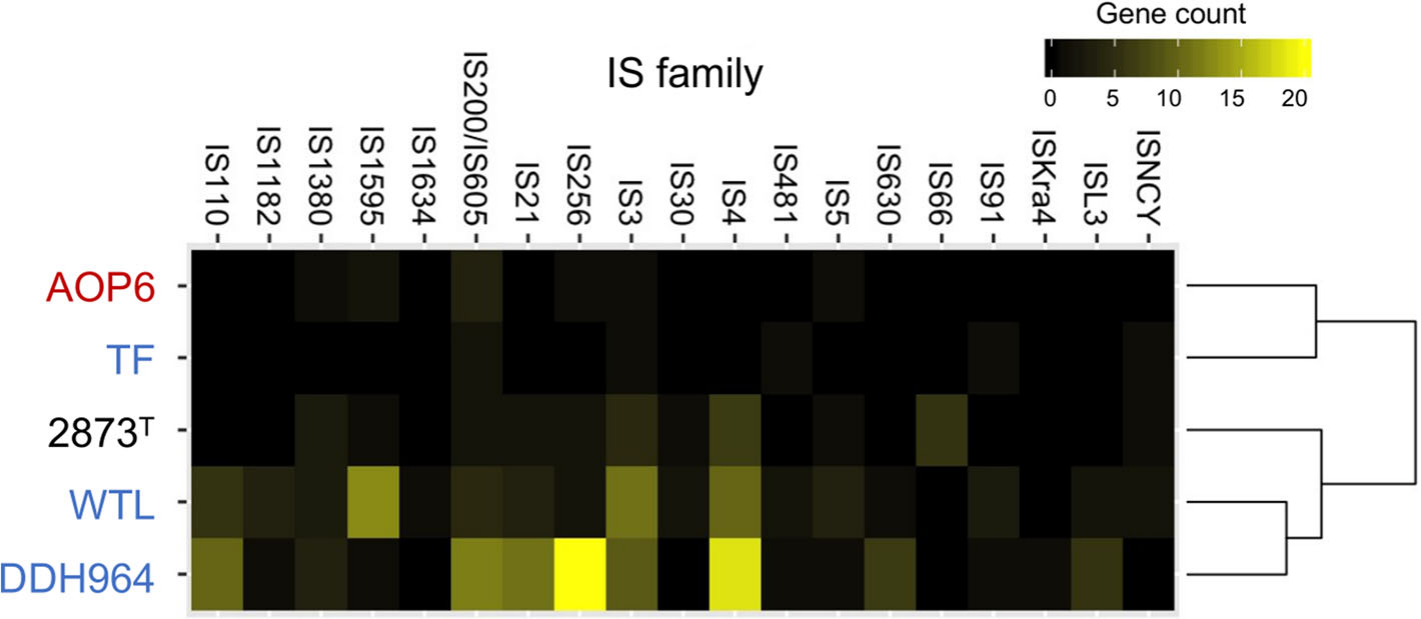
Heatmap with hierarchical clustering showing the insertion sequence (IS) families of the transposases detected in the 5 *Desulfuromonas* spp. strains. Red-, black-, and blue-colored strain names show those strains stimulated by using the crystalline Fe (III) oxide, sulfur and anode as the electron acceptors, respectively

In order to better understand the pattern of TEs, the predicted transposase genes were classified by IS family. This analysis showed that the transposase genes in the WTL genome were mainly affiliated to families IS*1595*, IS*3* and IS*4*, while those in the DDH964 genome were predominately from families IS*256*, IS*4*, IS*200*/IS*605*, IS*21*, IS*110*, IS*3*, and others (Fig. 6). The distinct profiles of IS family between the two genomes suggested that these anode-stimulated strains may have independently undergone the TE expansions. Our analysis identified the abundant and diverse transposons spread across the genomes of the anode-stimulated WTL and DDH964 (Figs. 3 and 6). Other important features were (i) the absence of the gene cluster for flagellar biosynthesis (see section 2) and (ii) the presence of diverse genes involved in oxygen resistance and ROS detoxification (see section 5) in these two genomes. We speculated that, when the flagellar gene cluster is lost from the *Desulfuromonas* genome, the bacterial mobility would be decreased and the cells would be restricted in specific habitats with relatively constant conditions, such as existing simple types of electron acceptors. This situation may trigger the transposon-mediated genome rearrangement that results in the selection of the essential genes for this specific habitat and the elimination of other unnecessary genes from the genome. However, the genome sizes of the strains WTL and DDH996 are larger than those of the strains AOP6 and 2873^T^, indicating the gene acquisition and enrichment may have also occurred during the genome rearrangement. It is likely that the decrease in mobility also affects the ability of escaping from the abrupt change in environment conditions, for example the exposure to oxygen, so that the genes involved in stress resistance would be gained and enriched in the genome. Interestingly, it was reported that the genes involved in the formation of pili and flagella, as well as chemotaxis sensory regulators, in *G. uraniireducens* were significantly down-regulated under oxidative stress conditions, while those for oxygen resistant enzymes were up-regulated under the same conditions [81], implying the opposite relationship between the expression of these genes. This evolutionary scenario partially explained that the strains AOP6 and 2873^T^ possessed more numerous numbers and types of the genes involved in chemotaxis and *c*-type cytochrome, while the strains WTL and DDH964 had more diverse genes for oxygen resistance.

## Conclusions

In this study, we carried out comparative genome analysis to identify the different genomic signatures between ferric iron oxide- and anode-stimulated *Desulfuromonas* spp. strains. The results indicated that the compared 5 genomes possessed distinct gene sets responsible for flagellar biosynthesis, chemotaxis, electron transfer, oxygen resistance and genome rearrangement. Particularly, the crystalline Fe (III) oxide-stimulated strain AOP6 would exhibit the flagellum-based mobility with more diverse chemotaxis sensory systems and more abundant *c*-type cytochromes for reduction of electron acceptors with low redox potentials. On the other hand, the anode-stimulated strains WTL and DDH964 lacking the flagellum-based mobility likely weakened the ability to use the low redox potential electron acceptors but enabled to survive under oxygen exposure. Moreover, the transposon expansions would mediate the genome rearrangements in the strains WTL and DDH964 genomes. These findings cast light on genome evolution of the phylogenetically close *Desulfuromonas* spp. strains that are involved in metal reduction in subsurface environments.

## Methods

### Genome-wide phylogeny of the order *Desulfuromonadales*

In order to perform a genome-wide phylogenetic analysis, all the available genomes (50 genomes in total) affiliated with the order *Desulfuromonadales* were obtained from GenBank/DDBJ/EMBL on March 11, 2020. Single copy marker genes were inferred using OrthoFinder v2.3.3 [91], and the amino acid sequences deduced from these genes were used to perform a multilocus sequence analysis as previously reported [84, 85], with minor modifications. In brief, multiple sequences alignments were inferred for each gene using MAFFT v7.455 [92] and concatenated for phylogenetic analysis. An approximately-maximum-likelihood phylogenetic tree was generated using FastTree v2.1.10 with the Gamma20 model [93]. FigTree v1.4.4 (https://github.com/rambaut/figtree) was used for the visualization of the tree.

### Comparative genomics

In an attempt to investigate the genomic features for the closely related *Desulfuromonas* spp. strains with different enrichment approaches, the complete genome sequences of the strains AOP6, WTL, and DDH964, as well as the draft sequences of strains 2873^T^ and TF, were selected for comparative genomic analysis (Table 1). In order to refine the previous annotations of these 5 genomes, the amino acid sequences deduced from all of the genes were analyzed using the KofamKOALA [94] and MOTIF searches against the sequence libraries Pfam (https://www.genome.jp/tools/motif), NCBI-CDD and PROSITE. Pairwise ANI and AAI among the 5 genomes were determined using the BLAST-based JspeciesWS [95] and online AAI calculator (http://enve-omics.ce.gatech.edu/aai/), respectively. The OGs across the 5 genomes were phylogenetically inferred using the OrthoFinder with the option of ‘diamond’, an accelerated sequence aligner that has a comparable sensitivity to the BLASTX. The presence or absence of genes was evaluated by determining whether or not the genome possessed the correspondent OG. A heatmap with hierarchical clustering based on the Bray-Curtis similarities on the R platform (https://www.r-project.org/) with the gplots package v.3.0.3 (https://cran.r-project.org/web/packages/gplots/index.html) was used to show the OG distribution across the 5 genomes. The predicted OGs were manually sorted based on the physical locations and directions in the 3 complete genomes to estimate the genomic difference and rearrangement. Circos v0.69 [96] was used to visualize the syntenic comparative analysis data of the AOP6 genome versus the WTL and DDH964 genomes.

Chemotaxis genes were identified using Microbial Signal Transduction Database (MiST) 3.0 [48]. The identified genes were sorted based on the orthologous groups to evaluate the presence or absence of the chemotaxis genes in each genome. The class membership of the MCP was assessed by the number of heptad repeats (H) in the cytoplasmic domain [50]. The organization of chemotaxis operons in the *Desulfuromoa*s spp. strains was predicted using FGENESB (http://www.softberry.com/berry.phtml) with the previously described algorithm [22]. Multiheme *c*-type cytochromes (> 3 Cxx [x] H motifs) were identified with the Python script reported previously [29]. The predicted multiheme *c*-type cytochromes were sorted based on the orthologous groups to generate a heatmap with hierarchical clustering mentioned above. The family membership of predicted transposases was assessed using ISfinder [97] and indicated as a heatmap with hierarchical clustering mentioned above. Pseudogenes were identified with the NCBI prokaryotic genome annotation pipeline employing a two-pass approach to detect frameshifted genes and pseudogenes with the premature stop codon [98].

## Supplementary Information

**Additional file 1.**

## Data Availability

All data analyzed in this study are available through NCBI GeneBank and RefSeq databases, are accessible through the accession numbers listed in Table 1 for the major data and in Table S1 for all the data, respectively.

## Abbreviations

AAI: Average Amino acid Identity
ANI: Average Nucleotide Identity
MCP: Methyl-accepting Chemotaxis Protein
OG: Orthologous Group
OGC: Orthologous Group of *c*-type Cytochrome
ROS: Reactive Oxygen Species
TE: Transposable Element
